# High apoptotic endothelial microparticle levels measured in asthma with elevated IgE and eosinophils

**DOI:** 10.1186/s12931-023-02470-x

**Published:** 2023-07-07

**Authors:** Yael Strulovici-Barel, Robert J. Kaner, Ronald G. Crystal

**Affiliations:** 1 Department of Genetic Medicine, Weill Cornell Medicine, 1300 York Avenue, Box 164, New York, NY 10065 USA; 2 Division of Pulmonary and Critical Care Medicine, Department of Medicine, Weill Cornell Medicine, New York, NY USA

**Keywords:** Airway remodelling, Angiogenesis, Biomarker, Pulmonary capillaries, Vascular remodelling

## Abstract

While asthma is considered an inflammatory-mediated airway epithelial and smooth muscle disorder, there is increasing evidence of airway capillary endothelial dysfunction associated with vascular remodelling and angiogenesis in some individuals with this condition. The inflammation is typically characterized as type-2 high (eosinophilic) vs type 2-low (neutrophilic and pauci-granulocytic); we hypothesized that the type-2 high group would be more likely to evidence endothelial dysfunction. As a biomarker of these processes, we hypothesized that nonsmokers with allergic asthma may have elevated plasma levels of endothelial microparticles (EMPs), membrane vesicles that are shed when endothelial cells undergo activation or apoptosis. Total and apoptotic circulating EMPs were measured by fluorescence-activated cell analysis in patients with allergic asthma (n = 29) and control subjects (n = 26), all nonsmokers. When the entire group of patients with asthma were compared to the control subjects, there were no differences in total circulating EMPs nor apoptotic EMPs. However, patients with asthma with elevated levels of IgE and eosinophils had higher levels of apoptotic EMPs, compared to patients with asthma with mildly increased IgE and eosinophil levels. This observation is relevant to precision therapies for asthma and highlights the importance of sub-phenotyping in the condition.

## Introduction

While most attention on the pathogenesis of asthma has focused on the role of airway epithelial inflammation and smooth muscle hypertrophy [1], asthma is also associated with enhanced airway wall angiogenesis and microvascular remodeling [2]. These airway vascular abnormalities are associated with increased blood flow, microvascular permeability and edema, contributing to the influx of inflammatory cells and airway narrowing. The increase in airway wall vascularity likely reflects a local increase in angiogenesis, an active process involving endothelial cell activation, proliferation and apoptosis [3]. Based on this background, we hypothesized that these changes in bronchial wall blood vessels in patients with asthma may be reflected by increased levels of circulating endothelial microparticles (EMPs), membrane vesicles that are shed when endothelial cells undergo activation or apoptosis [4]. Interestingly, the data demonstrates that a subset of patients with asthma with elevated levels of blood immunoglobulin (Ig) E and eosinophils have increased numbers of circulating EMPs derived from endothelial cells undergoing apoptosis.

## Methods

### Study population

Subjects were recruited under protocols approved by the Weill Cornell Medicine Institutional Review Board and provided written informed consent, as previously described [5]. The asthma group (n = 29) were all lifelong nonsmokers. All had evidence of reversible airflow obstruction and/or positive methacholine challenge, and a history of allergy with elevated serum IgE (> 165 IU/ml) and/or elevated blood eosinophils (> 0.45 × 10^3^/μl) and/or positive skin prick test to at least 1 common allergen. The non-allergic, healthy controls (n = 26), all lifelong nonsmokers, had normal serum IgE and blood eosinophil levels. All subjects underwent clinical assessment and evaluation of plasma EMPs.

### Endothelial microparticle analysis

As previously described [6], total numbers of circulating EMPs were characterized by fluorescence-activated cell analysis as 0.2 to 1.5 μm particles that were CD42b^−^CD31^+^ based on detection of CD42b (platelet glycoprotein Ib alpha chain) and CD31 (P-selectin, endothelial cell specific). To assess whether the circulating EMPs were derived from “activated” or “apoptotic” endothelial cells, measurement of CD62E (E-selectin) was added to the analysis, with elevated levels (compared to control subjects) of CD42b^−^CD62^+^/CD42b^−^CD31^+^ representing EMPs derived from “activated” endothelial cells and reduced levels (compared to control subjects) representing EMPs derived from “apoptotic” endothelial cells. P values comparing parameters between the groups were calculated using 2-sided Student’s t-test with unequal variance.

## Results

Control subjects were older and had a lower body mass index compared to the patients with asthma (p < 0.05, both comparisons, Table 1). Gender and ethnicity distributions were similar in both groups (p > 0.4, both comparisons). Serum IgE and blood eosinophils were significantly higher in patients with asthma *vs* control subjects (p < 0.002, both comparisons). Forced vital capacity (FVC) % predicted, forced expiratory volume in 1 s (FEV1) % predicted and FEV1/FVC were significantly lower, and % change in FEV1 post bronchodilator was significantly higher in patients with asthma compared to controls (p < 0.03, all comparisons).

**Table 1.** Demographics of the study population

Parameter	Controls	All asthma	Asthma with mild IgE and eosinophils^d^	Asthma with elevated IgE and eosinophils^e^	All asthma vs controls p value	Elevated vs mild allergic asthma p value
N	26	29	12	17		
Gender (M/F)	9/17	13/16	6/6	7/10	p > 0.4	p > 0.6
Age (yr)	37 ± 11	31 ± 11	31 ± 9	31 ± 12	**p < 0.05**^f^	p > 0.9
Race (B/W/H/O)^a^	12/7/4/3	15/5/7/2	8/1/2/1	7/4/5/1	p > 0.9	p > 0.7
Body mass index	26 ± 4	28 ± 5	29 ± 5	27 ± 5	**p < 0.04**	p > 0.2
Asthma severity (mild/moderate/severe)^b^	–	15/10/4	7/3/2	8/7/2	–	p > 0.8
IgE (IU/ml)	39 ± 38	627 ± 897	66 ± 32	1023 ± 1002	**p < 0.002**	**p < 0.003**
Absolute eosinophils (× 10^3^/μl)	0.2 ± 0.1	0.5 ± 0.4	0.2 ± 0.1	0.8 ± 0.3	**p < 10**^**–4**^	**p < 10**^**–4**^
Inhaled corticosteroids (on/not on)	–	9/20	3/9	6/11	–	p > 0.8
Pulmonary function parameters^c^						
FVC	112 ± 12	104 ± 16	103 ± 18	104 ± 15	**p < 0.03**	p > 0.9
FEV1	109 ± 11	87 ± 13	89 ± 12	85 ± 14	**p < 10**^**–8**^	p > 0.2
% change FEV1 post-bronchodilator	2.6 ± 2.8	11.4 ± 5.0	10.6 ± 2.7	11.9 ± 5.9	**p < 10**^**–4**^	p > 0.5
FEV1/FVC	81 ± 4	71 ± 9	73 ± 7	69 ± 11	**p < 10**^**–5**^	p > 0.2
TLC	103 ± 16	103 ± 18	99 ± 18	105 ± 17	p > 0.9	p > 0.3
DLCO	92 ± 10	88 ± 11	89 ± 10	87 ± 11	p > 0.2	p > 0.3

Data are presented as mean ± standard deviation, p values of numeric parameters calculated using a 2-tailed Student’s t-test with unequal variance, p value of categorical parameters calculated using a chi-square test

^a^B: Black; W: White; H: Hispanic; O: Other

^b^Mild: mild intermittent and mild persistent; moderate: moderate persistent; severe: severe persistent

^c^Pulmonary function testing parameters are given as % of predicted value, with the exception of FEV1/FVC, which is reported as % observed; *FVC* forced vital capacity, *FEV1* forced expiratory volume in 1 s, *TLC* total lung capacity, *DLCO* diffusing capacity

^d^ Mild IgE (≤165 IU/ml) and eosinophils (< 0.45 × 10^3^/μl)

^e^ Elevated IgE (>165 IU/ml) and eosinophils (≥ 0.45 × 10^3^/μl)

^f^ Threshold for significance: p < 0.05 (highlighted in bold)

**Fig. 1:**
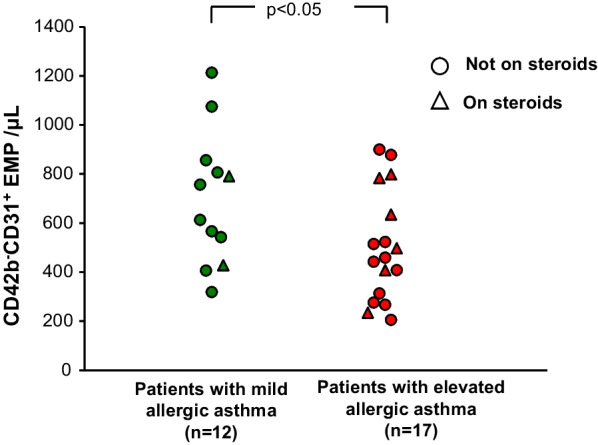
Comparison of total plasma endothelial microparticles (EMP) levels in patients with elevated (red) vs mild (green) allergic asthma. Circles = asthmatics not on steroid treatment, triangles = asthmatics on steroid treatment

**Fig. 2:**
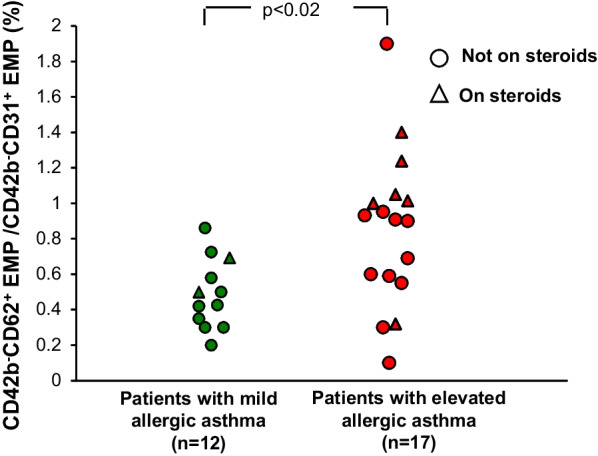
Comparison of activated plasma endothelial microparticles (EMP) levels in patients with elevated (red) *vs* mild (green) allergic asthma. As previously described [6], CD42b^−^CD62^+^/CD42b^−^CD31^+^ above normal controls are considered to be derived from “activated” endothelial cells while CD42b^−^CD62^+^/CD42b^−^CD31^+^ below normal controls are derived from “apoptotic” endothelial cells. Circles = asthmatics not on steroid treatment, triangles = asthmatics on steroid treatment

When the entire group of patients with asthma were compared to the controls, there were no differences in total circulating EMPs nor in activated or apoptotic EMPs (p > 0.6, both comparisons). However, since the process of increased bronchial wall angiogenesis and remodeling in asthma may only occur in a subset of patients with asthma [7], the study population of patients with asthma was divided into two groups based on IgE and eosinophils levels using the criteria: (1) a mildly allergic group with positive skin prick testing to ≥ 1 common allergen but lower IgE (≤ 165 IU/ml) and eosinophils (< 0.45 × 10^3^/μl), n = 12; and (2) a highly allergic group with positive skin prick testing to ≥ 1 common allergen and elevated IgE (> 165 IU/ml) and eosinophils (≥ 0.45 × 10^3^/μl), n = 17. These cutoffs were based on the upper limit of normal in our university hospital clinical laboratory, where the lab tests were run. Demographics and clinical characteristics including inhaled corticosteroid usage were similar between the asthma subgroups and there was no difference in the number of patients with mild, moderate or severe asthma within each group (p > 0.2, all comparisons). Compared to the mildly allergic group, the highly allergic group (referring to the severity of the allergy, not the clinical asthma symptoms) had significantly higher IgE (1023 ± 1002 IU/ml *vs* 66 ± 21 IU/ml, p < 0.003) and eosinophils (0.8 ± 0.3 × 10^3^/μl vs 0.2 ± 0.1 × 10^3^/μl, p < 10^–4^). Interestingly, while the patients with asthma with elevated IgE and eosinophil levels had less total EMPs compared to the patients with asthma with mild levels of IgE and eosinophils (509 ± 215/µl vs 698 ± 269/µl, p < 0.005; Fig. 1), they had elevated levels of apoptotic EMP as defined by a CD42b^−^CD62^+^/CD42b−CD31^+^ ratio lower than the mean observed in control subjects (0.49 ± 0.20 vs 0.85 ± 0.44, p < 0.03; Fig. 2), implying that there is active pulmonary capillary apoptosis ongoing. Within the subgroups of patients with asthma, there were no significant differences in total or activated/apoptotic EMPs whether they were on or were not on steroid treatment, or whether they had mild, moderate or severe asthma (p > 0.05, all comparisons). There was no correlation of total or apoptotic EMP levels with any of the demographic or lung function parameters (p > 0.09 and r^2^ ≤ 0.2, all parameters).

## Discussion

There is increasing evidence that some patients with asthma have airway wall vascular changes associated with airway remodeling [2]. The vascular abnormalities include increased numbers of capillaries in the bronchial wall and vascular remodeling, a process associated with vasodilation, capillary leak and edema, contributing to airway constriction [2]. As a correlate of this process, the present study identifies a subgroup of highly allergic patients with asthma with elevated plasma levels of EMPs derived from endothelial cells undergoing apoptosis.

Microparticles released by activated or apoptotic cells including the pulmonary capillary endothelium have been proposed as biomarkers in other chronic airway diseases [8]. Elevated apoptotic EMPs levels have been observed in smokers with low DLCO [6] and chronic obstructive pulmonary disease [9]. In asthma, it has been reported that platelet-derived microparticles are significantly elevated compared to healthy subjects [10]. In the same study, EMPs from the entire asthma population were unchanged, consistent with our observations in the patients with asthma were not sub-grouped by allergic-related criteria. Angiogenesis and microvascular remodeling have been linked to inflammation in asthma [2], and many inflammatory mediators including histamine, prostaglandins and leukotrienes contribute to angiogenesis, vasodilation and microvascular leakage, potentially leading to endothelial cell activation, dysfunction or apoptosis.

## Conclusions

In summary, assessment of plasma levels of apoptotic EMPs in nonsmoker patients with asthma suggests a novel endotype of highly allergic patients with asthma with elevated IgE and eosinophil levels having higher levels of apoptotic EMPs, compared to mildly allergic patient with asthma with lower IgE and eosinophil levels, an observation that should be further investigated in relevance to precision therapies for allergic asthma. Our analysis demonstrated difference in levels of apoptotic EMP in asthmatics with elevated levels of IgE and eosinophils compared to asthmatics with mild levels of IgE and eosinophils as endothelial cell activation or dysfunction could be considered as a potential therapeutic target in high allergic asthma. Importantly, circulating apoptotic EMPs are potential candidates as diagnostic or therapeutic biomarkers in highly allergic asthmatics, which might speed the development of new therapies specifically targeting this subgroup of asthmatics.

## Data Availability

Not applicable.

## Abbreviations

ACE2: Angiotensin converting enzyme 2
ANCOVA: Analysis of covariance
Controls: Nonsmoker healthy controls
COPD: Chronic obstructive pulmonary disease
COVID-19: Coronavirus disease-2019
FEV1: Forced expiatory volume in one second
ICS: Inhaled corticosteroids
ICS^+^: Nonsmoker asthmatics, treated with maintenance ICS
ICS^−^: Nonsmoker asthmatics, not treated with ICS
LAE: Large airway epithelium
SARS-CoV-2: Severe acute respiratory syndrome coronavirus 2
